# Short Communication: Novel Di- and Triselenoesters as Effective Therapeutic Agents Inhibiting Multidrug Resistance Proteins in Breast Cancer Cells

**DOI:** 10.3390/ijms25179732

**Published:** 2024-09-08

**Authors:** Dominika Radomska, Robert Czarnomysy, Krzysztof Marciniec, Justyna Nowakowska, Enrique Domínguez-Álvarez, Krzysztof Bielawski

**Affiliations:** 1 Department of Synthesis and Technology of Drugs, Medical University of Bialystok, Kilinskiego 1, 15-089 Bialystok, Poland; 2 Department of Organic Chemistry, Medical University of Silesia, Jagiellonska 4, 41-200 Sosnowiec, Poland; 3 Instituto de Química Orgánica General (IQOG-CSIC), Consejo Superior de Investigaciones Científicas, Juan de la Cierva 3, 28006 Madrid, Spain

**Keywords:** breast cancer, triple-negative breast cancer, anticancer drugs, selenium compounds, organoselenium compounds, selenoesters, cancer drug resistance, multidrug resistance, molecular docking, flow cytometry

## Abstract

Breast cancer has the highest incidence rate among all malignancies worldwide. Its high mortality is mainly related to the occurrence of multidrug resistance, which significantly limits therapeutic options. In this regard, there is an urgent need to develop compounds that would overcome this phenomenon. There are few reports in the literature that selenium compounds can modulate the activity of P-glycoprotein (MDR1). Therefore, we performed in silico studies and evaluated the effects of the novel selenoesters EDAG-1 and EDAG-8 on BCRP, MDR1, and MRP1 resistance proteins in MCF-7 and MDA-MB-231 breast cancer cells. The cytometric analysis showed that the tested compounds (especially EDAG-8) are inhibitors of BCRP, MDR1, and MRP1 efflux pumps (more potent than the reference compounds—novobiocin, verapamil, and MK-571). An in silico study correlates with these results, suggesting that the compound with the lowest binding energy to these transporters (EDAG-8) has a more favorable spatial structure affecting its anticancer activity, making it a promising candidate in the development of a novel anticancer agent for future breast cancer therapy.

## 1. Introduction

According to the GLOBOCAN database, there were nearly 2.3 million new cases of breast cancer (BC) and more than 666,000 deaths from it in 2022 alone [1]. One of the most disturbing facts is the ever-increasing mortality rate for this type of cancer. Its cause is believed to be fast-developing multidrug resistance (MDR) [2,3]. It is estimated that MDR occurs in about 90% of BC cases with fatal outcomes [2], which, according to the above data, affects nearly 600,000 people. This is a sufficient reason to intensify work on the development of new agents with anticancer activity for the treatment of BC that can overcome MDR.

Triple-negative breast cancer (TNBC) represents about one-fifth of all types of BC [4,5]. It is characterized by extreme aggressiveness, heterogeneity, and a high frequency of recurrence/metastasis formation, resulting in a significantly increased risk of treatment failure and a poorer patient prognosis. In addition, the absence of specific receptors on the BC cell surface (estrogen receptor (ER), progesterone receptor (PR), and human epidermal growth factor receptor 2 (HER-2)) results in the impossibility of molecularly targeted treatment [5,6,7], which significantly limits the possible therapies that can be applied. Unfortunately, this malignancy also exhibits rapid progression, and initially satisfactory therapeutic response to the applied treatment results in the development of chemoresistance in a relatively short time [6]. Therefore, the above features of TNBC result in more challenging treatment and a higher mortality rate than other BC subtypes [5].

Besides the absence of cell surface receptors, TNBC is associated with MDR [5,8]. Several factors contribute to the development of this unfavorable phenomenon, including epigenetics, the presence of cancer stem cells, the tumor microenvironment, and changes that affect the cell’s signaling pathways [9]. However, it has been found that the main cause of MDR is overexpression and dysregulation of the activity of membrane efflux pumps, mainly of the ABC (ATP-binding cassette) transporter family, which are responsible for transporting drugs into and out of the cell [8,9,10]. In the case of TNBC, the proteins most involved in the occurrence of MDR are MDR1 (also known as P-glycoprotein (P-gp), which causes resistance to therapy with cisplatin, doxorubicin (DOX), epirubicin, paclitaxel, docetaxel, etoposide, and vincristine), MRP1 (multidrug resistance protein 1, which causes resistance to treatment with anthracycline antibiotics, taxanes, methotrexate (MTX), and mitoxantrone), and BCRP (breast cancer resistance protein, which causes resistance to DOX, MTX, and 5-fluorouracil) [5,8]. Therefore, it is necessary to design substances that inhibit the activity of these transport proteins.

Specific inhibitors of individual ABC family transporters are used to inhibit the efflux of anticancer drugs, among others. Drugs belonging to the first generation of MDR1 inhibitors, such as verapamil or cyclosporin A, were not initially designed as molecules that inhibit this efflux pump, and their properties were discovered only after some time [11]. Verapamil is a calcium channel blocker used in heart diseases [12], and cyclosporine A is an immunosuppressive drug administered to transplant patients [13]. A common feature of these inhibitors is their low affinity for MDR1. In contrast, further generations of MDR1 inhibitors (second: biricodar, PSC 833, gallopamil; third: tariquidar, zosuquidar, and ontogen) are characterized by increasing affinity and binding potency to the MDR1 resistance protein (the strongest are exhibiting binding strengths of EC_50_ < 100 nM) [11,14]. In the case of compounds that inhibit the activity of the MRP1 efflux pump, it can be distinguished substances of plant origin (such as apigenin, curcumin, quercitin, or silymarin), antibodies (QCRL2/-3/-4, MIB6, Mab-IR700), tyrosinase inhibitors (ibrutinib, rapamycin and its analogs, rifampicin, dexamethasone), or other small molecules like MK-571 (considered one of the standard MRP1 transporter inhibitors) [15]. In addition, there is evidence that cyclosporin A also inhibits MRP1 and BCRP protein activity [15,16]. Meanwhile, many commonly used drugs for various diseases exhibit BCRP transporter inhibitory properties—these include ketoconazole or nitrendipine and their analogs, HIV (lopinavir) and HCV (telaprevir) protease inhibitors, and imatinib. Besides these, other highly selective BCRP inhibitors exist, which include fumitremorgin C and its derivatives [16]. Interestingly, tariquidar is also considered a BCRP transporter inhibitor [11,17]. Whereby, the most commonly used molecule with properties that inhibit the activity of the BCRP efflux pump is an antibiotic called novobiocin [16]. However, despite the wide range of compounds that modulate the activity of ABC transporters, there is still a search for agents that would bind even more potently and selectively to these resistance proteins.

Organic selenium (Se) compounds are a broad group of derivatives that have not yet been fully understood and described. The currently available worldwide literature evidences that these compounds are characterized by multitarget effects, and their anticancer activity is highly potent [18]. Moreover, some of the Se-compounds have been shown to exhibit MDR-overcoming activity via P-gp efflux pump inhibition [18,19], and these derivatives include selenoanhydride [20,21], phenylselenoethers [22], selenoflavones [23], and ethaselen [24], which, in turn, reverses cisplatin resistance in drug-resistant K562 leukemia cells.

Our long-term research [25,26] on Se-compounds showed that MDA-MB-231 (TNBC) cells are more sensitive to selenoesters than the MCF-7 cell line. We suspect these derivatives may inhibit MDR efflux pumps in TNBC cells. Therefore, given the above findings, we undertook an evaluation aimed at molecular docking and the ability for inhibition of BCRP, MDR1, and MRP1 resistance proteins by the novel selenoesters EDAG-1 and EDAG-8 in MCF-7 and MDA-MB-231 breast cancer cells.

## 2. Results

### 2.1. EDAG-1 and EDAG-8 Interact with BCRP, MDR1, and MRP1 Resistance Proteins in an In Silico Model (Molecular Docking)

**Figure 1:**
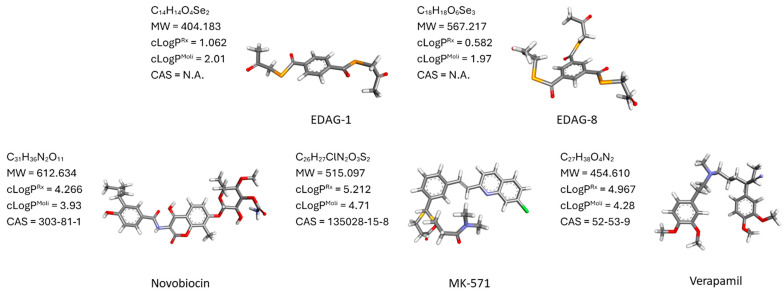
3D structures of the ligands used in the in silico study and their chemical formula, molecular weight, cLogP, and CAS number. Abbreviations: MW—molecular weight, Rx—calculated using Reaxys, Moli—calculated using Molispiration, N.A.—not available. See methodology for further details.

In the first stage of in silico research, optimization of the compounds EDAG-1, EDAG-8, and reference ligands (novobiocin, verapamil, and MK-571) was carried out using the Gaussian program. Additionally, the molecular weight of the tested compounds and their cLogP were calculated using the Reaxys chemistry database and the Molinspiration software (version 2024). Optimized structures of five ligands and their chemical formula, molecular weight, cLogP, and CAS number are shown in Figure 1.

Target proteins were pre-prepared for docking using Biovia software (version 19.1.0). In this program, water, metal ions, and cocrystallized ligands were removed from the crystallographic structures of proteins obtained from the PDB database. The resulting files (in pdb format) were then entered into AutoDock Vina. The preparation of files in the pdbq format was carried out by the default settings of the Vina program (adding hydrogen atoms, removing nonpolar hydrogen atoms, and adding partial charges using the Gasteiger–Marsili method).

The docking energy values for the obtained complexes are presented in Table 1. The lower the value of this energy, the greater the probability of binding the ligand to the binding site of the target protein. Analyzing Table 1, it can be observed that the lowest binding energy among all the tested complexes is exhibited by the selenoester EDAG-8, indicating that it has the highest binding potential with the resistance proteins BCRP, MDR1, and MRP1.

### 2.2. EDAG-1 and EDAG-8 as Inhibitors of ABC Transporters in Breast Cancer Cells

**Figure 2:**
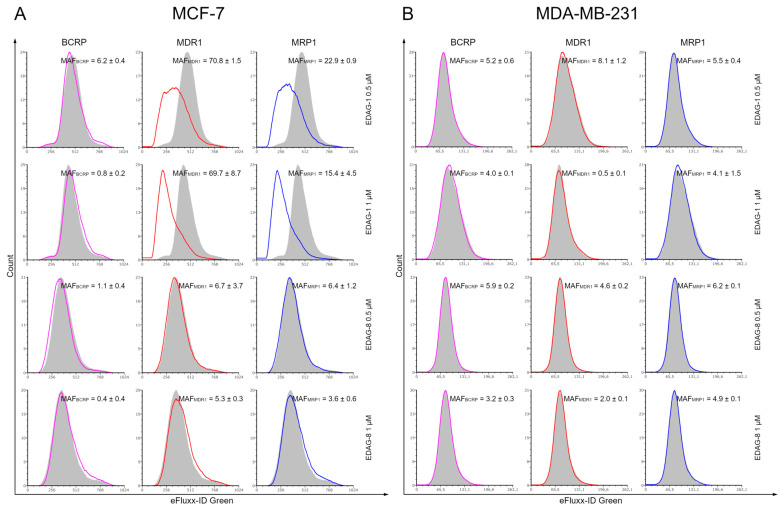
Cytometric analysis of the BCRP, MDR1, and MRP1 resistance protein activities in MCF-7 (**A**) and MDA-MB-231 (**B**) breast cancer cells exposed for 24 h to the selenoesters EDAG-1 and EDAG-8. The tested breast cancer cells were incubated with Efflux Green Detection Reagent and the appropriate inhibitors (novobiocin, verapamil, and MK-571). The gray-filled histogram presents the fluorescence of cells not exposed to the inhibitor, while the pink, red, and blue histograms represent cells treated with the BCRP, MDR1, and MRP1 inhibitors, respectively. The difference in mean fluorescence intensity (MFI) indicates the corresponding ABC transporter activity. The MDR activity factors (MAF) with SD (standard deviation) in the upper-right corner quantify characteristics related to multidrug resistance.

The present study was designed to evaluate the effects of the tested selenoesters on BCRP, MDR1, and MRP1 resistance proteins in MCF-7 and MDA-MB-231 breast cancer cells after 24 h of incubation with them at concentrations of 0.5 and 1 µM. The general principle of this study is that the more the tested compound inhibits the pumping out of Efflux Green Detection Reagent (EGDR) by ABC transporters, the more it is retained inside the cell, and MAF values are lower. According to the cytometric measurements, the autofluorescence of the culture medium and tested breast cancer cells (MCF-7 and MDA-MB-231) was insignificant, as it was less than 2% relative to the tested samples (MFI varied between 4 and 8; see Supplementary Materials) and was therefore omitted from the calculations. Analyzing Figure 2, it can be observed that EDAG-1 and EDAG-8 compounds inhibit resistance proteins in most cases (MAF < 20), and their effectiveness depends on the concentration of the tested derivative. In the case of the selenoester EDAG-1, it was noted that at a concentration of 0.5 µM, it did not inhibit the activity of MDR1 and MRP1 proteins (MAF values were 70.8 and 22.9, respectively) in MCF-7 cells, and at a concentration of 1 µM, this phenomenon occurred only in MDR1 (MAF = 69.7). Meanwhile, at a concentration of 1 µM, this selenoester inhibited the MRP1 pump, and the MAF value was equal to 15.4. Lower efflux of EGDR by MDR1 and MRP1 transporters under the influence of EDAG-1 was observed in MDA-MB-231 triple-negative breast cancer cells (MAF = 8.1 and 0.5 (MDR1) as well as 5.5 and 4.1 (MRP1) for concentrations of 0.5 and 1 µM, respectively). In turn, compound EDAG-8 inhibited tested efflux pumps in both cell lines at each concentration (Figure 2). For MDR1 and MRP1 pumps, MAF values were 6.7 and 6.4 (0.5 µM concentration), as well as 5.3 and 3.6 (1 µM concentration) for the MCF-7 cell line, respectively, while for MDA-MB-231 cells they were 4.6 and 2.0 (0.5 µM concentration), as well as 6.2 and 4.9 (1 µM concentration). Inhibition of BCRP protein occurred both in MCF-7 and MDA-MB-231 cells at the two concentrations of each tested compound, and MAF values were as follows: 6.2 (EDAG-1 0.5 µM), 0.8 (EDAG-1 1 µM), 1.1 (EDAG-8 0.5 µM), and 0.4 (EDAG-8 1 µM) for MCF-7; and 5.2 (EDAG-1 0.5 µM), 4.0 (EDAG-1 1 µM), 5.9 (EDAG-8 0.5 µM), and 3.2 (EDAG-8 1 µM) for MDA-MB-231. In summary, in most cases, MAF values were lower in MDA-MB-231 cells than in MCF-7 cells. In summary, the tested derivatives appear to be inhibitors of ABC transporters. In addition, in most cases, MAF values were lower in MDA-MB-231 triple-negative cells than in MCF-7 cells.

## 3. Discussion

Breast cancer is one of the most common malignancies worldwide. It is characterized by a high mortality rate, which is primarily related to rapidly developing chemoresistance [1,2,3]. To reverse this trend and lower the death rate, there is a need for novel molecules with anticancer activity that would overcome MDR.

Studies on Se and its compounds indicate that derivatives containing this element in their structure are characterized by chemopreventive and anticancer activity. Selenoesters are a group of compounds with potent anticancer properties, but they are not yet well understood [18,27]. Our previous studies [25] reveal that MDA-MB-231 triple-negative breast cancer cells are more sensitive to this group of compounds, which could suggest the potential overcoming of resistance in these cells.

**Figure 3:**
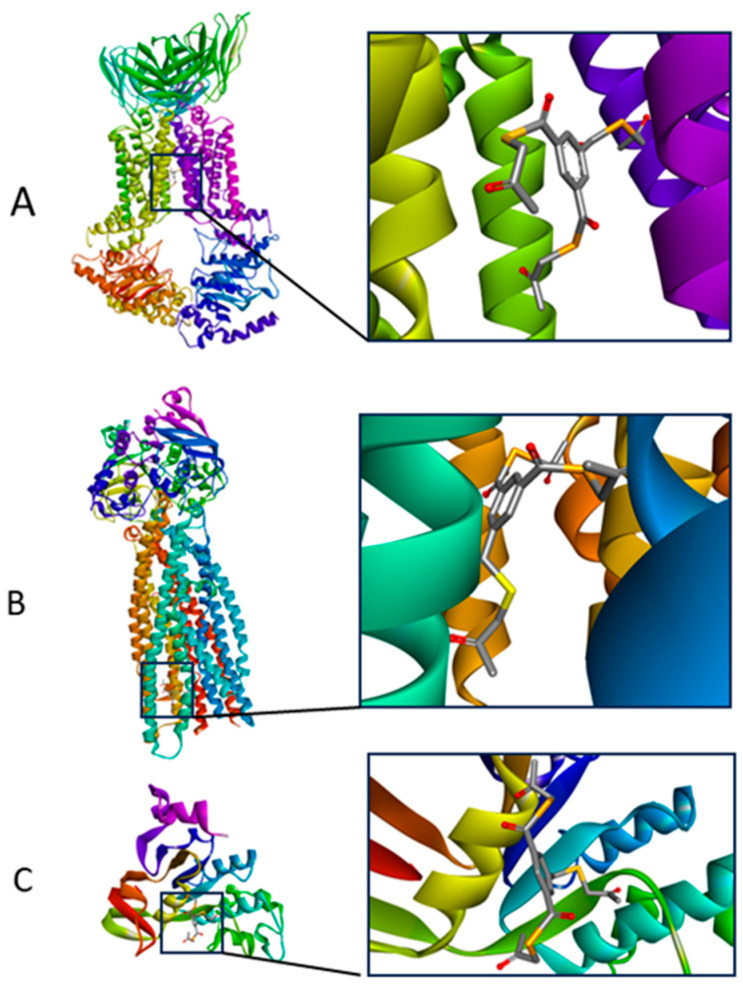
The lowest-energy docking poses of compound EDAG-8 with BCRP (**A**), MDR1 (**B**), and MRP1 (**C**) proteins.

**Figure 4:**
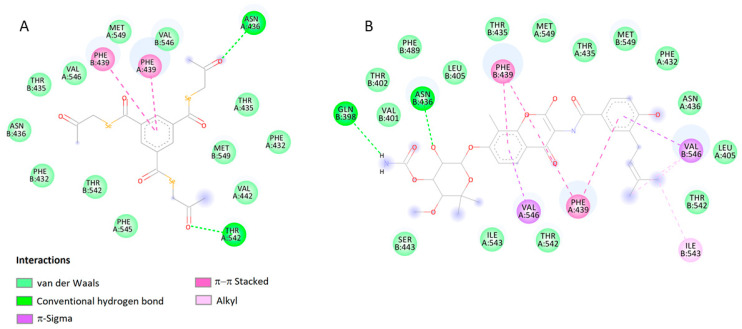
2D visualization of the interaction between EDAG-8 (**A**), novobiocin (**B**), and BCRP protein.

**Figure 5:**
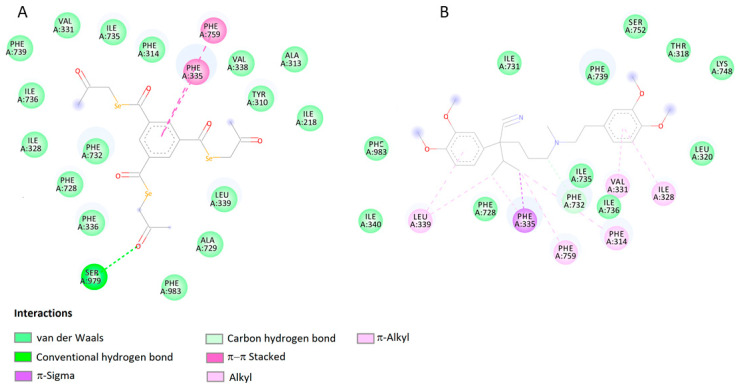
2D visualization of the interaction between EDAG-8 (**A**), Verapamil (**B**), and MDR1 protein.

**Figure 6:**
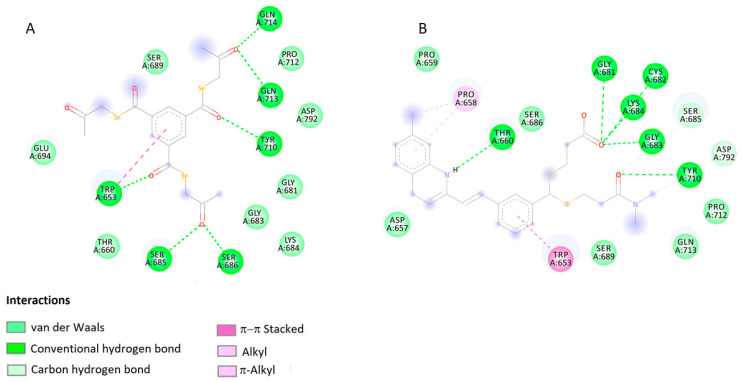
2D visualization of the interaction between EDAG-8 (**A**), MK-571 (**B**), and MRP1 protein.

The binding energy values of selected ligands with target proteins obtained as a result of docking indicate that the compound EDAG-8 has the highest binding potential. Higher than the selected reference ligands (MK-571, verapamil, and novobiocin). It should also be mentioned that the docking score values for the compound EDAG-1 also indicate a high modulatory potential of this derivative, especially toward the MRP1 protein (Table 1). Figure 3 shows the lowest-energy docking poses of derivative EDAG-8 with BCRP, MDR1, and MRP1 proteins. A detailed analysis of the impacts of the selenoester EDAG-8 is presented in Figure 4, Figure 5 and Figure 6 and Table 2.

Analyzing the interactions of the compound EDAG-8 with the BCRP protein (Figure 4A, Table 2), two strong hydrogen bonds are visible between the ligand ketone groups and ASN436 and THR542 located in the A chain of the protein’s binding site. Furthermore, the benzene ring of the ligand generates π–π staking interactions with the benzene ring of PHE439 located in the A and B chains of the protein. The arrangement of the compound EDAG-8 in the binding pocket of the BCRP protein is similar to the spatial arrangement of the reference novobiocin (Figure 4B). In this complex, a hydrogen bond is visible between the ligand and ASN436. PHE439 located in the A and B chains of the BCRP protein is also involved in the stabilization of the complex.

A similar pattern of interaction is visible in the complex of EDAG-8 and the MDR1 protein (Figure 5A). One of the ketone groups of the ligand forms a hydrogen bond with the protein binding site SER979, and the benzene ring interacts through π–π stacking interactions with the benzene rings of two phenylalanine residues (PHE335 and PHE759). When comparing the verapamil nature of the interactions at the target protein (Figure 5B), it should be emphasized that they are only hydrophobic. There are no stronger hydrogen bond interactions in the obtained complex.

In particular, numerous interactions are visible between the ligand and the MRP1 protein (Figure 6A). The complex shows six hydrogen bonds generated by the carbonyl oxygen atoms of the ligand and the amino acids of the protein binding site (TRP653, SER685, SER686, TYR710, GLN713, and GLN714). Additionally, π–π stacking interactions are visible between the aromatic rings of the ligand and TRP653. A similar pattern of interactions is observed between the target protein and the reference compound MK571 (Figure 6B). The MK571 and MRP1 protein is also stabilized by six hydrogen bonds and is additionally stabilized by π–π stacking interactions. This nature of the interactions, similar to the interactions in the EDAG-8 selenoester, shows the high modulatory potential of the EDAG-8 derivative.

The stability of all three complexes obtained in silico studies is stabilized by numerous van der Waals interactions.

In summary, it can be stated that the molecular docking results indicate a high modulatory potential of the selenoester EDAG-8. Furthermore, they also showed that this compound may belong to the group of highly potent but relatively nonspecific ABC transporter inhibitors. The derivative EDAG-1 may also have such a potential.

The prevalence of multidrug resistance (MDR) is a very unfavorable phenomenon in many fields of medicine, including oncology. Lack of adequate drug action against cancer cells leads to ineffectiveness of the applied therapy, which forces clinicians to use ever higher doses of anticancer substances. Unfortunately, this is often associated with severe toxicity against normal cells and thus the occurrence of dangerous side effects. Among the main reasons for the development of MDR in cancer cells is the excessive efflux of drugs by pumps belonging to ATP-dependent transporters, which include breast cancer resistance protein (BCRP), P-glycoprotein (P-gp, MDR1), and multidrug resistance protein 1 (MRP1) [5,8]. Recent reports indicate that some Se-compounds can inhibit P-gp activity more than the reference verapamil in colorectal cancer [23] or T-lymphoma [22], among others. However, to date, there is no report on the activity of Se-containing compounds against BCRP and MRP1 proteins. Considering this literature and cognitive gap, as well as the greater sensitivity of MDA-MB-231 triple-negative breast cancer cells to selenoesters [25,26], we evaluated the effects of this group of compounds on ABC transporters, i.e., BCRP, MDR1, and MRP1. Our results suggest that selenium derivatives have a more potent inhibitory effect on the MDR1 transporter than verapamil, confirming reports previously published by other authors [18,22,23,28]. Moreover, as observed, in most cases, MAF values were lower in MDA-MB-231 cells than in MCF-7 cells, with the selenoester EDAG-8 appearing to be more effective as an inhibitor of resistance proteins. The higher modulatory potential of this compound may be due to the spatial conformation of the molecule, and thus its lower binding energy (compared to EDAG-1 or conventionally used inhibitors, i.e., novobiocin, verapamil, and MK-571), as well as better matching of the ligand to the efflux pumps tested.

Summarizing the above, the present study revealed that the tested selenoesters may be efficient inhibitors of ABC transporters in breast cancer cells. It is also worth noting that their activity is double-action—anticancer (potent cytotoxicity with IC_50_ in the nanomoles range [26]), along with simultaneous inhibition of resistance proteins. Indeed, our previous studies [25,26] indicate that a group of these Se compounds exhibit anticancer activity as a result of the induction of apoptosis and autophagy in breast cancer cells, which provides a promising framework for the development of anticancer agents with simultaneous nullifying activity on the efflux of them from cancer cells. In addition, a significant future perspective would be to investigate whether the presence of selenoesters (e.g., EDAG-8) can enhance the therapeutic efficacy of conventionally used anticancer drugs (combination therapy) to which resistance develops. Increased sensitivity to anticancer drugs of resistant cells could indicate their ability to overcome this phenomenon and their use as potential adjuvants in BC therapy in the future.

## 4. Materials and Methods

### 4.1. Materials

Dimethyl sulfoxide (DMSO) was purchased from Sigma-Aldrich (St. Louis, MO, USA). Stock cultures of human breast cancer cells (MCF-7 and MDA-MB-231) were obtained from the American Type Culture Collection (ATCC, Manassas, VA, USA). Dulbecco’s Minimal Eagle Medium (DMEM), fetal bovine serum (FBS), phosphate-buffered saline (PBS) used in cell culture, trypsin, glutamine, penicillin, and streptomycin were provided from Gibco (San Diego, CA, USA). MDR Assay Kit was delivered by Abcam (Cambridge, UK).

### 4.2. Tested Compounds

**Figure 7:**
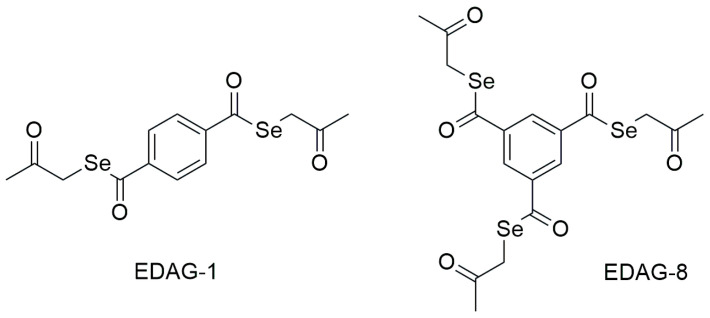
Structural formulas of the tested Se-compounds (selenoesters).

Two Se-compounds were investigated—EDAG-1 [*Se,Se*-bis(2-oxopropyl) benzene-1,4-bis(carboselenoate)] and EDAG-8 [*Se,Se,Se*-tris-(2-oxopropyl) benzene-1,3,5-tris-(carboxyselenoate)] (Figure 7). These compounds have a ketone end fragment and two or three selenoester moieties in their molecule. Their detailed synthesis and chemical characterization have been described in a patent application [29].

### 4.3. Molecular Docking of EDAG-1 and EDAG-8

The three-dimensional structures of EDAG-1, EDAG-8, and references novobiocin, verapamil, and MK-571 were optimized and energy minimized using Gaussian 16 (rev. A.03) computer code [30] using the density functional theory (DFT, B3LYP) and 6311+G(d,p) basis sets. Selected target proteins for molecular docking studies were obtained from the Protein Data Bank (https://www.rcsb.org/, accessed on 28 July 2024). We used the crystal structure of BCRP (PDB ID: 7NEQ), MRP1 (PDB ID: 2CBZ), and MDR1 (PDB ID: 6C0V). The AutoDock Vina [31] tool compiled in PyRx [32] was used for the docking analysis. The region of interest used for Au-to-Dock Vina docking was defined as X = 126.37, Y = 126.5, Z = 120.9 for BCRP, X = −20.13, Y = 46.95, Z = 6.16 for MRP1, and X = 164.7, Y = 145.32, Z = 196.5 for MDR1. The volume was set at 25 × 25 × 25 Å. After calculations, only the 9 highest-scored poses were returned as a docking result for the ligand-cavity configuration. All the results obtained were presented in kilocalories per mol. Preliminary preparation of proteins and molecular docking details were visualized using the BIOVIA Discovery Studio virtual environment [33].

Reaxys, a database of chemical compounds built by Elsevier, was used to calculate molecular weight and cLogP, by drawing the compounds in the online software of the database. Additionally, to compare values with different software, Molinspiration online software (version 2024) was also used to provide a cLogP value by drawing the compounds in the online tool of the application. Molispiration is a private company of cheminformatics (Molinspiration Cheminformatics) founded as a spin-off of Bratislava University (Slovak Republic). The significant discrepancy in cLogP values found for selenocompounds is normal and may be caused by a non-fully correct parametrization of selenium in these software tools. A prior study of the group found a significant variation between the predicted data for specific selenocompounds by different software applications and the data experimentally obtained at the laboratory [34]. 

### 4.4. Cell Culture of MCF-7 and MDA-MB-231 Breast Cancer Cells

Human breast cancer cell lines (MCF-7 and MDA-MB-231) were purchased from the American Type Culture Collection (ATCC, Manassas, VA, USA). MCF-7 and MDA-MB-231 cells were cultured in Dulbecco’s Modified Eagle Medium complemented by 10% of fetal bovine serum (FBS) and 1% of antibiotics: penicillin and streptomycin (all from Gibco, San Diego, CA, USA). The cells were maintained in an incubator that provides the optimal growth conditions for the cell culture: 5% CO_2_, 37 °C, and humidity in a range of 90-95%. The cells were cultured in 100 mm plates (Sarstedt, Newton, NC, USA). Subsequently, after obtaining a subconfluent cell culture, the cells were detached with 0.05% trypsin with 0.02% EDTA (Gibco, San Diego, CA, USA). Then, a Scepter 3.0 handheld automated cell counter (Millipore, Burlington, MA, USA) was used for quantifying the number of cells that were subsequently seeded at a density of 5 × 10^5^ cells per well in six-well plates (“Nunc”) in 2 mL of the growth medium. In this study, cells that obtained 80% confluence were used.

### 4.5. Multidrug Resistance (MDR) Proteins Activity Assay

The effect of the tested compounds on the activity of MDR transporters in MCF-7 and MDA-MB-231 breast cancer cells (baseline seeding density: 5 × 10^5^ cells/well—manufacturer recommendation) was evaluated after 24 h of incubation with them at concentrations of 0.5 and 1 µM (rounded mean IC_50_ and 2 x IC_50_ values obtained in the MTT assay from our recent study [26]). The MDR Assay kit (Abcam, Cambridge, UK) was used for this purpose. The most significant component included in this kit is Efflux Green Detection Reagent—a substrate for the tested ABC transporters (BCRP, MDR1, MRP1). When resistance proteins are not actively pumping out this reagent, it is degraded by intracellular esterases, causing the dye to be retained inside the cell. Therefore, the mean fluorescence intensity (MFI) depends on the activity of the transporter proteins present in the cell membrane and is inversely proportional to their activity. The lower the activity of the resistance proteins, the more dye is retained in the cell, and the MFI is higher. The entire assay was conducted following the manufacturer’s instructions available on their website. After 24h, the cells were collected and washed twice with PBS (2 × 5 mL) by centrifugation (1200 rpm, 10 min). Further, the supernatant was removed, and the cells were counted utilizing a Scepter 3.0 handheld automatic cell counter (Millipore, Burlington, MA, USA). Then, the cells were resuspended (density 2 × 10^6^ cells/mL) in a pre-warmed culture medium (Dulbecco’s Modified Eagle Medium, Gibco, San Diego, CA, USA), and each sample was divided into four parts (250 µL each). Diluted inhibitor or medium containing 5% DMSO was added to each tube according to the scheme: 1A—125 µL MDR1 inhibitor (verapamil), 1B—125 µL MRP1 inhibitor (MK-571), 1C—125 µL BCRP inhibitor (novobiocin), 1D—125 µL medium/5% DMSO (sample without inhibitor), 2A—125 µL MDR1 inhibitor (verapamil), 2B—125 µL MRP1 inhibitor (MK-571), etc. Baseline concentrations of each used inhibitor were 80 µM, 200 µM, and 400 µM (concentrations imposed by the kit manufacturer) for verapamil, MK-571, and novobiocin, respectively. Such prepared samples were incubated for 5 min in an incubator providing optimal growth conditions for cell culture (5% CO_2_, 37 °C, and 90–95% humidity). After the required incubation time, 125 µL of Efflux Green Detection Reagent (previously diluted in the medium at the 1:50 ratio) was added to each sample, pipetted (avoiding the introduction of bubbles), and incubated again for 30 min under the same conditions as before. After 25 min of incubation, 5 µL of propidium iodide (250 µg/mL) was added to each tube and incubated for the remaining 5 min. Following this time, thus prepared samples were transferred to cytometric tubes and immediately analyzed using a flow cytometer (BD FACSCanto II; 10,000 events measured; flow rate 100–300 events/sec) with FACSDiva 6.0 software (both from BD Biosciences Systems, San Jose, CA, USA), and then with FCS Express 7 software (De Novo Software, Pasadena, CA, USA). Additionally, the autofluorescence levels of the culture medium alone (without cells; DMEM) and of MCF-7 and MDA-MB-231 breast cancer cells were also measured (the original autofluorescence histograms of culture medium and breast cancer cells from flow cytometry analysis, see Supplementary Materials). The equipment was calibrated with BD Cytometer Setup and Tracking Beads (BD Biosciences, San Diego, CA, USA).

The multidrug resistance activity factor (MAF) was calculated separately for each transporter. The calculations considered the difference between the MFI of cells with and without inhibitors (medium containing 5% DMSO). The MAF values were calculated using the formulas listed below:

$${MAF}_{MDR1}=100\times \frac{\left|F_{MDR1}-F_{0}\right|}{F_{MDR1}}$$

$${MAF}_{MRP1}=100\times \frac{\left|F_{MRP1}-F_{0}\right|}{F_{MRP1}}$$

$${MAF}_{BCRP}=100\times \frac{\left|F_{BCRP}-F_{0}\right|}{F_{BCRP}}$$

**F_MDR1_**—MFI with MDR1 inhibitor (verapamil)
**F_MRP1_**—MFI with MRP1 inhibitor (MK-571)
**F_BCRP_**—MFI with BCRP inhibitor (novobiocin)
**F_0_**—MFI without inhibitor (5% DMSO)

According to the manufacturer’s annotation, the theoretical range of MAF values is between 0 and 100. The manufacturer suggests that samples with MAF values < 20 are considered multidrug resistance negative, and the tested drug inhibits the respective ABC transporter. Meanwhile, MAF values > 25 indicate multidrug resistance positive, and the tested drug does not inhibit the respective ABC transporter.

**Figure 8:**
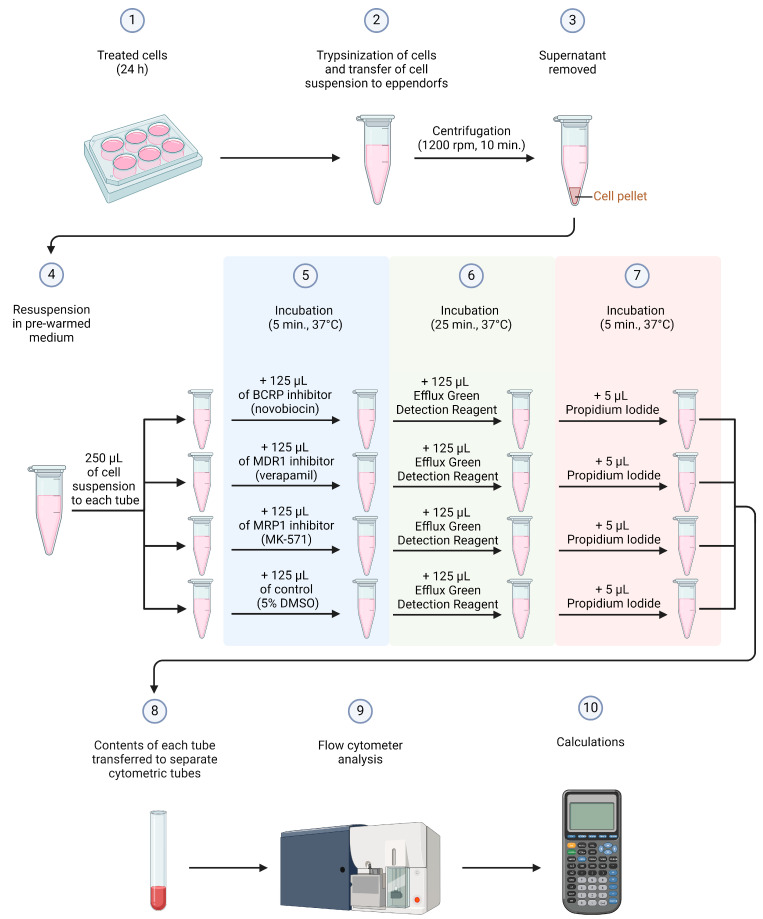
General scheme for multidrug resistance proteins activity assay.

The general scheme summarizing the above research method is shown in Figure 8.

## 5. Conclusions

In this work, we evaluated the effect of the novel selenoesters EDAG-1 and EDAG-8 on inhibiting resistance proteins in MCF-7 and MDA-MB-231 breast cancer cells. Breast cancer resistance protein (BCRP), P-glycoprotein (P-gp, MDR1), and multidrug resistance protein (MRP1), belonging to the family of ATP-binding cassette (ABC) transporters, were analyzed. The binding energy of the tested compounds to these transporters was determined in silico, and then the compound with the lowest binding energy (EDAG-8) was docked in these proteins. Hydrogen bonds and π–π stacking interactions are involved in the binding of this derivative, and the structure is stabilized by numerous van der Waals interactions. Meanwhile, flow cytometer analysis of the BCRP, MDR1, and MRP1 efflux pump activities indicated the potential of the tested compounds, especially the selenoester EDAG-8, to modulate these transporters. The main premise from both studies is that the derivative EDAG-8 is a more potent inhibitor of efflux pumps in breast cancer cells than the reference compounds (novobiocin, verapamil, and MK-571). In conclusion, the above results suggest that compound EDAG-8 has a more favorable spatial structure affecting its inhibitory activity of ABC transporters (BCRP, MDR1, and MRP1) than the derivative EDAG-1. Therefore, it may serve as a promising candidate in the development of a novel anticancer agent for breast cancer therapy in the future.

## Figures and Tables

**Table 1:** Calculated AutoDock Vina binding energies of tested compounds.

Compound	∆G kcal/mol		
	BCRP	MDR1	MRP1
**EDAG-1**	−9.1	−7.9	−6.7
**EDAG-8**	−10.7	−10.1	−7.3
**Novobiocin**	−10.5	-	-
**Verapamil**	-	−9.0	-
**MK-571**	-	-	−7.1

**Table 2:** Detailed interactions of EDAG-8 ligand and reference ligands with selected proteins.

Protein		Ligand		Interaction	
Name	Chain:Residue	Name	Residue	Type	Distance [Å]
**BCRP**	A:ASN436	EDAG-8	carbonyl oxygen	conventional hydrogen bond	2.37
	A:THR542		carbonyl oxygen	conventional hydrogen bond	1.99
	A:PHE439		benzene ring	π–π stacked	3.66
	B:PHE439		benzene ring	π–π stacked	4.54
	B:ASN436	novobiocin	hydroxyl group	conventional hydrogen bond	2.83
	B:GLN398		amine group	conventional hydrogen bond	2.63
	A:VAL546		benzene ring	π-sigma	3.70
	B:VAL546		benzene ring	π-sigma	3.91
	A:PHE439		benzene ring	π–π stacked	5.00
	A:PHE439		chromone ring	π–π stacked	5.51
	B:PHE439		benzene ring	π–π stacked	3.87
	B:PHE439		chromone ring	π–π stacked	5.17
	B:ILE543		butene chain	alkyl	4.46
	B:VAL546		butene chain	alkyl	4.61
	B:VAL546		butene chain	alkyl	3.79
**MDR1**	A:SER979	EDAG-8	carbonyl oxygen	conventional hydrogen bond	2.37
	A:PHE335		benzene ring	π–π stacked	4.35
	A:PHE759		benzene ring	π–π stacked	5.48
	A:PHE732	verapamil	*n*-alkyl group	carbon-hydrogen bond	3.67
	A:PHE335		isopropyl group	π-sigma	3.58
	A:LEU339		isopropyl group	alkyl	4.56
	A:PHE314		isopropyl group	π-alkyl	5.07
	A:PHE335		isopropyl group	π-alkyl	4.17
	A:PHE759		isopropyl group	π-alkyl l	4.17
	A:LEU339		benzene ring	π-alkyl	5.03
	A:ILE328		benzene ring	π-alkyl	5.07
	A:VAL331		benzene ring	π-alkyl	5.48
**MRP1**	A:TRP653	EDAG-8	carbonyl oxygen	conventional hydrogen bond	2.35
	A:SER685		carbonyl oxygen	conventional hydrogen bond	2.86
	A:SER686		carbonyl oxygen	conventional hydrogen bond	2.03
	A:TYR710		carbonyl oxygen	conventional hydrogen bond	2.56
	A:GLN713		carbonyl oxygen	conventional hydrogen bond	2.67
	A:GLN714		carbonyl oxygen	conventional hydrogen bond	1.82
	A:TRP653		benzene ring	π–π stacked	4.66
	A:GLY681	MK-571	carboxyl group	conventional hydrogen bond	2.72
	A:CYS682		carboxyl group	conventional hydrogen bond	2.90
	A:GLY683		carboxyl group	conventional hydrogen bond	2.07
	A:LYS684		carboxyl group	conventional hydrogen bond	2.27
	A:LYS684		carboxyl group	conventional hydrogen bond	2.49
	A:TYR710		amide group	conventional hydrogen bond	2.63
	A:THR660		endocyclic nitrogen atom	conventional hydrogen bond	2.22
	A:SER685		amide group	carbon hydrogen bond	3.52
	A:ASP792		*n*-methyl group	carbon-hydrogen bond	3.64
	A:TRP653		benzene ring	π–π stacked	3.76
	A:PRO658		methyl group	alkyl	4.33
	A:PRO658		benzene ring	π-alkyl	4.44

## Data Availability

Data are contained within the article or supplementary material. The original contributions presented in the study are included in the article/supplementary material, further inquiries can be directed to the corresponding author/s.

## Supplementary Materials

The following supporting information can be downloaded at: https://www.mdpi.com/article/10.3390/ijms25179732/s1.
