# Supplementary material for: Short Communication: Novel Di- and Triselenoesters as Effective Therapeutic Agents Inhibiting Multidrug Resistance Proteins in Breast Cancer Cells
Source: Int J Mol Sci. 2024 Sep 8;25(17):9732. doi: 10.3390/ijms25179732 (PMC11395623; doi:10.3390/ijms25179732)
Supplement: Supplementary file 1 [file ijms-25-09732-s001.zip › ijms-3156760-supplementary.pdf]

Supplementary Materials

Short Communication: Novel Di- and Triselenoesters as Effective Therapeutic Agents Inhibiting Multidrug Resistance Proteins in Breast Cancer Cells

Dominika Radomska [1*], Robert Czarnomysy [1*], Krzysztof Marciniec [2], Justyna Nowakowska [2], Enrique Domínguez-Álvarez [3] and Krzysztof Bielawski [1]

[1] Department of Synthesis and Technology of Drugs, Medical University of Bialystok, Kilinskiego 1, 15-089 Bialystok, Poland; dominika.radomska@umb.edu.pl (D.R.); robert.czarnomysy@umb.edu.pl (R.C.)
[2] Department of Organic Chemistry, Medical University of Silesia, Jagiellonska 4, 41-200 Sosnowiec, Poland
[3] Instituto de Química Orgánica General (IQOG-CSIC), Consejo Superior de Investigaciones Científicas, Juan de la Cierva 3, 28006 Madrid, Spain
* Correspondence: dominika.radomska@umb.edu.pl (D.R.); robert.czarnomysy@umb.edu.pl (R.C.); Tel.: +48-85-748-57-00; Fax: +48-85-879-57-00

This document contains representative histograms from the flow cytometric analysis, which show the level of measured autofluorescence of the culture medium and the tested cancer cells – their mean fluorescence intensity (MFI).

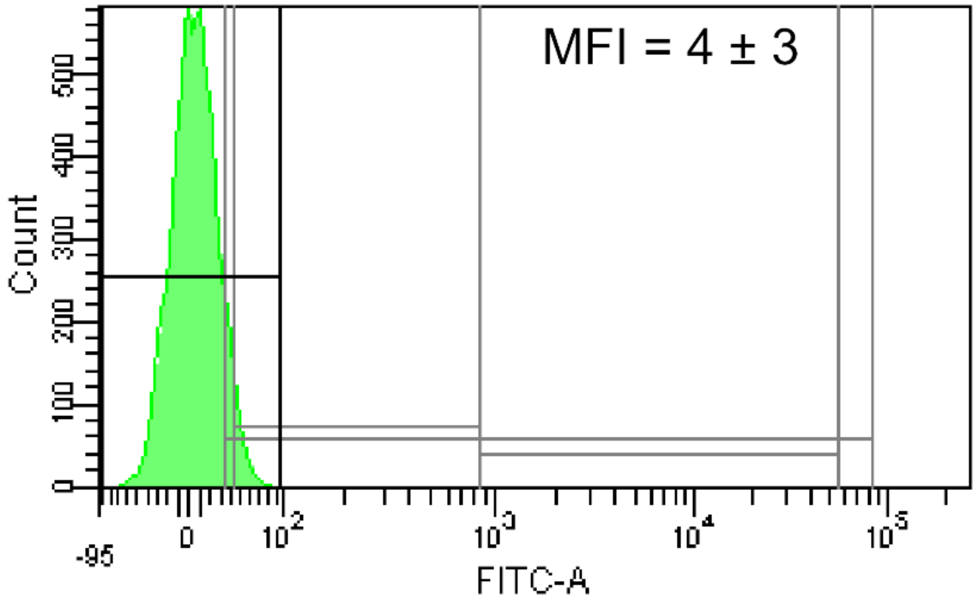

Figure S1. The original histogram from the cytometric analysis that was utilized to assess the level of autofluorescence in the culture medium used in the study (Dulbecco's Minimal Eagle Medium; DMEM). The mean values with standard deviation were reported based on data from three separate experiments (n = 3) conducted in triplicate.

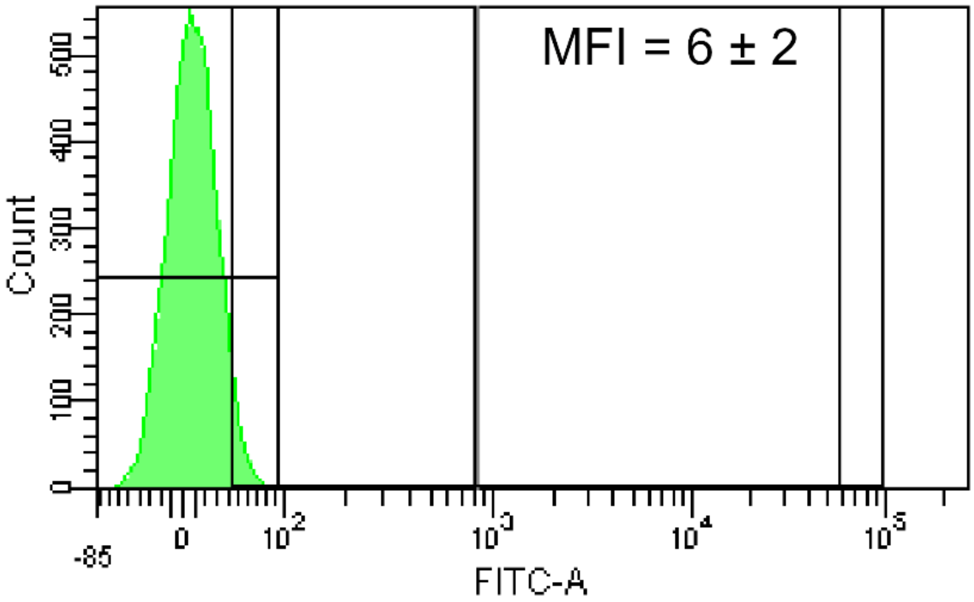

Figure S2. The original histogram from the cytometric analysis that was utilized to assess the level of autofluorescence in MCF-7 breast cancer cells. The mean values with standard deviation were reported based on data from three separate experiments (n = 3) conducted in triplicate.

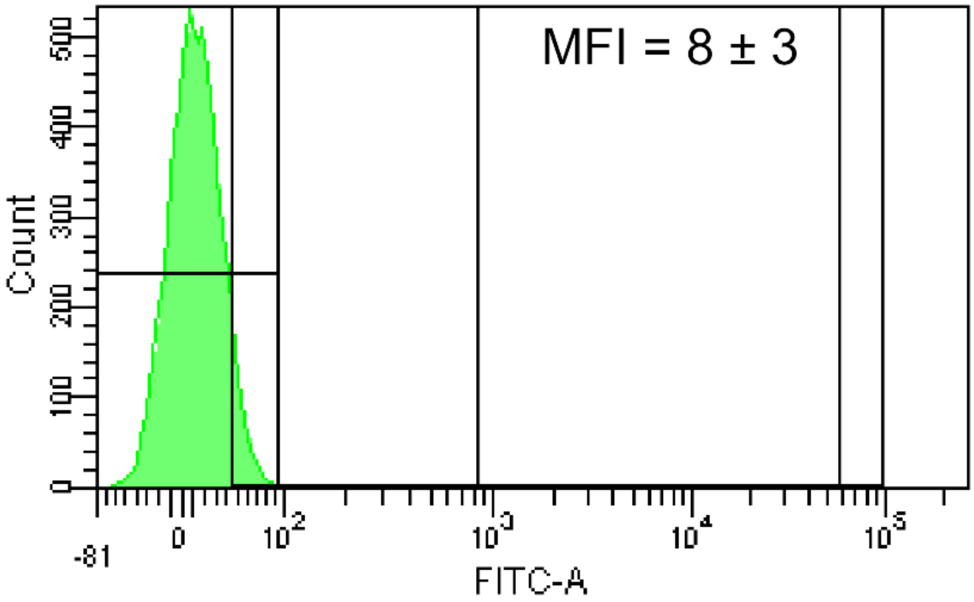

Figure S3. The original histogram from the cytometric analysis that was utilized to assess the level of autofluorescence in MDA-MB-231 breast cancer cells. The mean values with standard deviation were reported based on data from three separate experiments (n = 3) conducted in triplicate.
